# Clonal Strain Persistence of *Candida albicans* Isolates from Chronic Mucocutaneous Candidiasis Patients

**DOI:** 10.1371/journal.pone.0145888

**Published:** 2016-02-05

**Authors:** Alexander J. Moorhouse, Claire Rennison, Muhammad Raza, Desa Lilic, Neil A. R. Gow

**Affiliations:** 1 Aberdeen Fungal Group, School of Medical Sciences, Institute of Medical Sciences, University of Aberdeen, Aberdeen, United Kingdom; 2 Institute of Cellular Medicine, Faculty of Medical Sciences, Newcastle University, Newcastle upon Tyne, United Kingdom; Leibniz Institute for Natural Products Research and Infection Biology- Hans Knoell Institute, GERMANY

## Abstract

Chronic mucocutaneous candidiasis (CMC) is a primary immunodeficiency disorder characterised by susceptibility to chronic *Candida* and fungal dermatophyte infections of the skin, nails and mucous membranes. Molecular epidemiology studies of CMC infection are limited in number and scope and it is not clear whether single or multiple strains inducing CMC persist stably or are exchanged and replaced. We subjected 42 C. albicans individual single colony isolates from 6 unrelated CMC patients to multilocus sequence typing (MLST). Multiple colonies were typed from swabs taken from multiple body sites across multiple time points over a 17-month period. Among isolates from each individual patient, our data show clonal and persistent diploid sequence types (DSTs) that were stable over time, identical between multiple infection sites and exhibit azole resistant phenotypes. No shared origin or common source of infection was identified among isolates from these patients. Additionally, we performed *C*. *albicans* MLST SNP genotype frequency analysis to identify signatures of past loss of heterozygosity (LOH) events among persistent and azole resistant isolates retrieved from patients with autoimmune disorders including CMC.

## Introduction

Chronic mucocutaneous candidiasis (CMC) is a primary immunodeficiency disorder characterised by susceptibility to *Candida* spp. infection of the skin, nails and mucous membranes. It manifests separately to vulvovaginal candidiasis and rarely as invasive candidiasis. Generally presenting as chronic localised lesions, CMC persists due to isolated primary immune defects or secondary to predisposing immune compromising conditions such as HIV. It can also present as a heterogeneous disorder such as autoimmune polyendocrinopathy candidiasis ectodermal dystrophy (APECED), as a multisystem syndrome with hyper immunoglobin E syndrome (HEIS) or hypothyroidism, and can be complicated by additional autoimmune conditions such as inflammatory bowel disease (IBD) [1–3].

In healthy individuals, immunological surveillance of potential invasive microorganisms involves immune recognition of the pathogen and activation of innate and adaptive immune responses [4]. In CMC patients, defects affecting innate (dectin-1, CARD9, IL12RB1) and adaptive (interleukin (IL)17-F, IL17 receptor, STAT1, STAT3) immunity disrupt the protective mucosal Th-17 pathway preventing effective response to superficial fungal infections [1]. The largest aetiological class of CMC is caused by a dominant mutation in STAT1 [5,6] such as gain of function at coil-coiled domain [3] and DNA binding domain [1,7]. A premature stop mutation in the dectin-1 gene is also a known risk factor for CMC with allele prevalence of 3–8% in healthy populations [8]. Detailed discussion of CMC aetiology benefits from several informative reviews [1,9,10]. Understanding the molecular epidemiology of *Candida* spp. in chronic fungal infection is vital to developing improved preventative and therapeutic treatments and strategies to alleviate suffering. DNA marker typing strategies for *C*. *albicans* isolates have been explored extensively [11–13], and multilocus sequence typing (MLST) is now a standard measure of population structure for the major pathogenic *Candida* spp. [14–20]. Notably, MLST studies of non-*C*. *albicans*, *Candida* spp. reveals a more marked clonality among molecular types with a highly stable population structure in *C*. *dubliniensis* [21–23], *C*. *glabrata* [24,25], and *C*. *tropicalis* [26,27]—commonly encountered *Candida* spp. after *C*. *albicans*.

The accepted model of *C*. *albicans* population structure is one of predominant clonality with rare recombination events as a result of cryptic mating strategies generating new highly differential strain types, but more often, incremental mutation events such as point mutations and loss of heterozygosity (LOH) responsible for low level, within population, genetic diversity and adaptation [28–31]. The prevailing stability of *C*. *albicans* populations is observed most pertinently in studies using isolates taken at serial time points, which also provides opportunity to observe the emergence of antifungal drug resistant phenotypes and associated mutational changes when they do arise [32–34].

Molecular epidemiology studies have targeted *Candida* spp. population structure within nosocomial infections [35,36], among drug resistant isolates [37,38], and specific infection types [39–42], often seeking to observe the extent of particular *Candida* spp, clades and strain type enrichment within cohorts [43,44]. As yet few studies have addressed these epidemiological patterns among isolates causative of CMC, a condition in which chronic infections may persist beyond several years [45,46]. Two previous studies investigating the molecular epidemiology of CMC patients demonstrate the emergence of azole resistant *C*. *albicans* DSTs causative of infection. McMannus *et al*. conducted serial swab sampling and identified 18 *C*. *albicans* DSTs from 14 CMC patients. Multiple DSTs identified from individual patients were as a result of micro-variation rather than strain replacement or recombination. They also demonstrated acquisition of azole resistance over time as a result of mutations in *TAC1* and *ERG11* [46]. Siikala *et al*. [45] also conducted serial sampling of 9 CMC patients and observe similarly persistent infection along with mutations in *TAC1* and *ERG11* genes. Interestingly these mutations did not always confer increased resistance to azoles and were occasionally transient within infection populations. Jacobsen *et al*. [47] identified a reduced number of DSTs among non-serial oral isolates with autoimmune conditions including Lichen Planus, Sjorgen’s syndrome and mucous membrane pemphigoid when compared with healthy volunteers and VVC patients. Here we report clonal and persistent DSTs in CMC patients over a 17 month period, and evidence of loss of heterozygosity in persistent colonising strains.

## Materials and Methods

### Patient Recruitment, Background and Swabbing

More than 20 patients with chronic mucocutaneous candidiasis (CMC) were followed in our regional “Candidiasis” clinics. We report only on six unrelated patients, four females (P1, 2, 4 and 6) and two males (P3 and 5) for whom we found persistent positive cultures. Swabs were taken from infected sites (mouth, genitals, skin) for diagnostic culture and sensitivity testing to confirm *Candida* presence. Patients P1-P5 had a primary immune deficiency (PID) manifesting as CMC. Patient P6 had secondary oral candidiasis as a consequence of using steroid inhalers for severe asthma. We previously assessed all PID CMC patients by whole exome sequencing as part of a separate study [3] in which we identified gain-of-function STAT1 mutations in P1, P3 and P5, no mutation was identified in P4 or P6. GOF-STAT1 CMC patients and P6 had disease onset in early childhood/adolescence while P4 who had a candida granuloma of the soft palate and has since developed squamous cancer of the same area first presented at 50 years of age. PID CMC patients were followed for at least 5 years in this clinic, during which time all of them were on antifungal oral treatments. Antifungals were selected based on sensitivities and were either azoles (fluconazole and itraconazole) or amphotericin suspension 100 mg/ml (produced in our local hospital Pharmacy), diluted 1:10 before taking into mouth, swirled and swallowed 4x daily. (See Table 1).

**Table 1:** Patient clinical data. Flucon = fluconazole, itracon = itraconazole, amph sus = amphotericin suspension (10 mg/ml swirl and swallow 4x daily). All swabs were taken for routine Dg purposes. Ethical approval was obtained under the Newcastle Autoimmune Inflammatory Rheumatic Disease Research Biobank (NAIRB) Ref No NAIRB-DL-01 dx obtained from the Southwest 3 Research Ethics Committee (Ref. 10/H0106/30) to perform research on samples collected as part of the NAIRB by researchers based at Newcastle University. Pts 1–5 had primary CMC; whole exome sequencing confirmed gain-of-function STAT1 mutations in 3 while no mutation was identified in 2 patients; P4 had candida granuloma of soft palate. P6 had 2oCMC due to steroid inhalers (asthma). Swabs were processed in the Department of Microbiology, Newcastle upon Tyne Hospitals NHS Trust (NUTH): Mohammad Raza, Consultant Microbiologist (Muhammad.Raza@nuth.nhs.uk) and Claire Rennison, Senior BMS (now retired).

Pt No	M/F	age	Diagnosis	onset of CMC	Swab date	*Candida* spp. Identified	Prescribed
1	F	64	1oCMC (GOF-STAT1)	adolescence	14/11/2011	*C*. *albicans* + *C*. *nivariensis*	Flucon 100 mg/d
					16/04/2012	*C*. *albicans*	Itracon 100 mg/d
					09/07/2012	*C*. *albicans*	Flucon 100 mg/d
					14/01/2013	*C*. *albicans*	Flucon 100 mg/d
2	F	42	1oCMC (GOF-STAT1)	adolescence	13/02/2012	*C*. *albicans*	Flucon 100 mg/d
3	M	43	1oCMC (GOF-STAT1)	early childhood	09/07/2012	*C*. *albicans*	Flucon 100 mg/d
					10/09/2012	*C*. *albicans*	Itracon 100 mg/d
					12/11/2012	*C*. *albicans*	Itracon 100 mg/d
					13/01/2014	*C*. *albicans*	Itracon 100 mg/d + amph sus 10 mg/ml x4/d
4	F	61	1oCMC (granuloma)	10 yrs ago	13/02/2012	*C*. *albicans*	Flucon 100 mg/d;7 days each month
5	M	30	1oCMC (GOF-STAT1)	early childhood	14/05/2012	*C*. *albicans*	Flucon 100 mg/d
6	F	43	2o CMC (steroid inhalers)	8yrs ago	12/11/2012	*C*. *albicans*	Itracon 100 mg/d
					08/03/2013	*C*. *albicans*	amph sus 10 mg/ml x4/d

Multiple colonies were typed from swabs taken over a 17 month period. Patients 1 and 3 were swabbed 4 times, patient 6 was swabbed twice and patients 2, 4 and 5 were swabbed once. Oral swabs were taken at all sampling time points, swabs of the groin and buttocks were taken for the penultimate time point of patient 3 (Table 1 and S1 Fig).

### Isolation and species identification

Patient samples were plated and purified on Sabouraud dextrose (Sabdex) agar plates containing 1% mycological peptone (w/v), 4% (w/v) glucose and 2% (w/v) agar and CAN2 agar (Biomerieux) to detect the presence of mixed isolates. Isolates of yeasts were also inoculated into Sabdex broth. Species identification was carried out using MALDI TOF mass spectrometry. All swabs were taken for routine diagnostics purposes. Ethical approval was obtained under the Newcastle Autoimmune Inflammatory Rheumatic Disease Research Biobank (NAIRB) Ref No NAIRB-DL-01 dx obtained from the Southwest 3 Research Ethics Committee (Ref. 10/H0106/30) to perform research on samples collected as part of the NAIRB by researchers based at Newcastle University.

### Antifungal susceptibility testing

Minimum inhibitory concentrations were determined by broth micro-dilution testing using a modification of the CLSI (formerly NCCLS) guidelines M27-A2 [48]. Drug concentrations ranged from 0.032 mg/L to 16 mg/L for caspofungin and a non-liposomal amphotericin B suspension, and 0.13 mg/L to 64 mg/L for fluconazole and voriconazole. Each drug was serially diluted with sterile water to 50 μL in flat bottomed 96 well plates. Cultures were grown in NGY medium (0.1% (w/v) neopeptone, 0.4% (w/v) glucose, 0.1% (w/v) yeast extract) for 12 h at 30°C to exponential phase and 20 μL transferred to 11 mL RPMI inoculation medium (2x RPMI-1640, 2% (w/v) glucose, buffered to pH 7.0 with MOPS). Aliquots of 50 μL inocula in RPMI were added to drug plates (total volume 100 μL), incubated for 24 h at 37°C and analysed using VERSAmax microplate reader at 405 nm. Each of 42 sequenced colonies were assayed in triplicate, and MIC90 and MIC50 values reported.

### Genomic DNA extraction and sequencing

Genomic DNA was extracted from yeast grown from single colonies in overnight YPD broth cultures (1% yeast extract, 2% bacto-peptone and 1% glucose). Pelleted cultures were subject to mechanical lysis in 2 mL Eppendorf tubes with 200 μl extraction buffer (100mM Tris-HCl [pH 8.0] containing 2% Triton X-100, 1% sodium dodecyl sulfate, and 1 mM EDTA), 200 μl 1:1 (vol/vol) phenol chloroform and 0.3g of acid washed glass beads (0.45 to 0.52 mm in diameter) using a bead beater. Addition of 200 μL TE (1 mM EDTA, 10 mM Tris-HCl, pH 8.0) preceded phase separation by centrifugation and ethanol precipitation of DNA containing upper phase, as previously described [18]. For each single colony isolate the internal region of seven housekeeping genes were amplified using seven pairs of primers (S1 Table) in separate PCR reactions with a first cycle of denaturation for 2 min at 94°C, followed by 25 cycles of denaturation at 94°C for 1 min, annealing at 52°C for 1 min, elongation at 72°C for 1 min, and a final extension step of 10 min at 72°C. PCR reactions were performed in 20 μl volumes using high-fidelity DNA polymerase (Finnzymes Phusion F-530L), with thermal cycler conditions as advised by the manufacturer and as previously described [16]. Aliquots of 5 μl of PCR products were run on agarose gels to confirm successful generation of products at expected sizes and 5 μl aliquots were purified using Mag-Bind E-Z magnetic beads as per manufacturer’s instructions. Chain-terminating dideoxynucleotide Sanger sequencing reactions were prepared from purified PCR products separately for forward and reverse strands and precipitated with addition of 15 μl sterile H2O to prevent dye blobs, 50 μl 3 M NaOAC (pH 5.2) followed by 70% ethanol rinse as previously described [15,49]. PCR products containing purified and dried sequencing reactions were sent to the University of Oxford, Department of Zoology and sequenced on ABI 3730 instruments. DNA sequence results were analysed and SNPs determined using DNASTAR Lasergene SeqMan Pro software. Strain types were further confirmed by subsequent re-sequencing using genomic DNA freshly prepared from cultures grown from -80°C glycerol strain stocks. DSTs were submitted to the *C*. *albicans* MLST central curation database http://pubmlst.org/calbicans/.

### Phylogenetic Analysis

Sequence data at previously identified *C*. *albicans* MLST SNP positions were extracted from our data and concatenated for multiple sequence alignment using Clustal Omega and visualised in MATLAB R2012a. Tree building was carried out with the Unweighted Pair Group Method with Arithmetic Mean (UPGMA) and visualised using FigTree v1.4.0. Splits phylogenies were prepared in SplitsTree4 v4.13.1. eBurst analysis was performed with eBURSTv3 [50]. (S2 and S3 Figs).

### Datasets

A non-redundant dataset of 2867 isolates (Dataset A) was prepared from the centrally curated MLST *C*. *albicans* database as at December 2014 (This database is presently in the process of migrating from http://calbicans.mlst.net/ to http://pubmlst.org/calbicans/); DST entries were not included if they were a repetition of the same DST from the same patient unless they occurred at a separate body site. Further datasets were chosen and constructed from the in house database of isolates; a reference that has been used to compile and update the online curated database since its inception. Dataset B: 245 individual isolates of various carriage types (9 VVC, 77 commensal, 13 candidaemia, 5 sepsis, 38 candiduria and 103 isolates of unspecified origin), Dataset C: the present data from 42 sequenced colonies from 6 CMC patients, Dataset D: 36 sextuple sequenced colonies from 6 VVC patients, Dataset E: 24 sextuple sequenced colonies from 4 autoimmune patients with Lichen Planus, Sjogren’s syndrome, mucous membrane pemphigoid and Lichen Planus respectively, Dataset F: 34 individual isolates from blood stream infection, Dataset G: 36 sextuple sequenced colonies of oral origin from 6 students. These datasets were chosen as several series of isolates present in the database as blocks of consecutive isolates submitted as discrete collections that have formed part of previously published MLST work [30,47,51]. (S4 Fig).

### SNP frequency distribution

At all SNP positions across MLST sequences, implementing the IUPAC ambiguity nomenclature for heterozygous SNPs, the frequency of each SNP genotype were calculated for Dataset A and the frequency distribution matrix was used to prepare normalised frequency distribution matrices of the 6 further datasets (B-G). Further data reduction was performed by tallying within non-overlapping 10% ranges and multiplying these counts by the range upper bound value to produce a normalised frequency distribution (NFD) of normalised frequency presence (NFP), and absence (NFA) in respect of heterozygous (NFPhet, NFAhet) and homozygous (NFPhom and NFAhom) classes of SNPs. (see also S5 and S6 Figs).

## Results

MLST analysis was performed on 42 *C*. *albicans* isolates from 6 CMC patients collected over a period of 17 months. All isolates retrieved from the same patient had the same DST and no LOH, point mutations or larger scale genetic differences were observed between colonies obtained from the same patient. Of the 6 DSTs observed, only one (DST 392) had been previously reported. These data show *C*. *albicans* DSTs persisting across time, between different anatomical sites as identical DSTs mutually exclusive for each patient in this cohort (Table 2 and S2 Fig).

**Table 2:** MLST DSTs for 42 *C*. *albicans* isolates from 6 CMC patients and their antifungal susceptibilities.

Patient Swabs and Isolates					MLST Results									Antifungal Susceptibility (MIC50 | MIC90)			
Patient	swab ref	swab date	N	Site	AAT1a	ACC1a	ADP1	MPIb	SYA	VPS13	ZWF1b	DST	Clade	caspofungin	amphoteracin B	fluconazole	voriconazole
Patient 1 (*N* = 13)	G967972M	11/14/2011	4	mouth	2	2	5	2	2	24	12	2121	1	0.0625 | 0.125	0.0625 | 0.125	64 | 64	2 | 16
	G93496H	4/16/2012	3	mouth	2	2	5	2	2	24	12	2121	1	0.0625 | 0.125	0.0625 | 0.125	32 | 64	8 | 16
	G165439G	7/9/2012	3	mouth	2	2	5	2	2	24	12	2121	1	0.0625 | 0.0625	0.0625 | 0.25	32 | 64	8 | 16
	G317213R	1/14/2013	3	mouth	2	2	5	2	2	24	12	2121	1	0.0625 | 0.0625	0.125 | 0.25	64 | 64	8 | 16
Patient 2 (*N* = 1)	AAZNR457	11/16/2011	1	mouth	4	7	4	4	4	244	4	2125	2	0.015625 | 0.25	0.125 | 0.25	8 | 32	0.5 | 4
Patient 3 (*N* = 19)	W991978S	12/12/2011	4	mouth	107	12	21	5	6	4	22	2127	11	0.125 | 0.25	0.125 | 0.25	32 | 64	32 | 64
	W165365R	7/9/2012	3	mouth	107	12	21	5	6	4	22	2127	11	0.25 | 0.25	0.125 | 0.25	32 | 64	32 | 64
	W214304P	9/10/2012	3	mouth	107	12	21	5	6	4	22	2127	11	0.25 | 0.25	0.25 | 0.25	32 | 64	32 | 64
	W317091H	1/14/2013	3	groin	107	12	21	5	6	4	22	2127	11	0.25 | 0.25	0.125 | 0.25	32 | 64	32 | 64
	W317092W	1/14/2013	3	buttock	107	12	21	5	6	4	22	2127	11	0.25 | 0.25	0.125 | 0.25	32 | 64	32 | 64
	W317093P	1/14/2013	3	mouth	107	12	21	5	6	4	22	2127	11	0.125 | 0.25	0.125 | 0.25	32 | 64	8 | 64
Patient 4 (*N* = 4)	L41606D	2/13/2012	4	mouth	8	3	8	4	7	10	8	392	4	0.015625 | 0.015625	0.0625 | 0.25	16 | 32	0.125 | 0.25
Patient 5 (*N* = 1)	E126427K	5/14/2012	1	mouth	4	7	14	4	134	4	4	2129	2	0.015625 | 0.015625	0.0625 | 0.125	16 | 64	0.25 | 0.25
Patient 6 (*N* = 4)	S267505Y	11/12/2012	1	mouth	14	7	8	4	7	3	8	2130	4	0.0625 | 0.125	0.0625 | 0.125	8 | 32	4 | 8
	S387147Q	3/8/2013	3	mouth	14	7	8	4	7	3	8	2130	4	0.03125 | 0.125	0.0625 | 0.125	8 | 16	4 | 8

### Phylogenetic analysis

**Fig 1:**
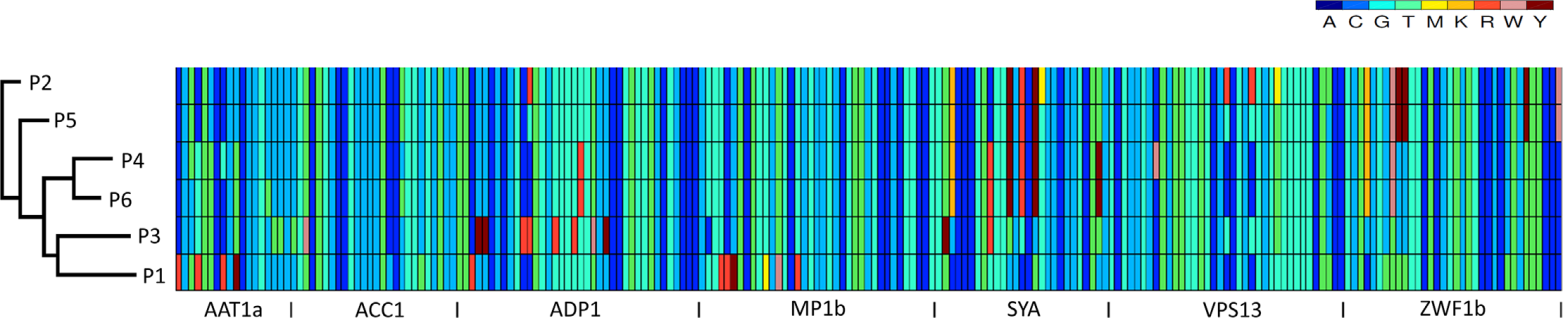
Multiple sequence alignment of concatenated SNPS of DSTs from 6 CMC patients. Multiple sequence alignment of C. albicans MLST concatenated SNPs for the 6 DSTs. Blue and green colours are homozygous SNPs, yellow, orange, red, pink and brown are heterozygous SNPs, the boundaries of the 7 sequenced regions are indicated at the x axis. SplitsTree phylogram of the DSTs profiles appears at the y axis.

Phylogenetic neighbour net analysis of DST profiles together with multiple sequence alignment and UPGMA tree of concatenated SNP positions demonstrated the distinct genetic backgrounds of these isolates. Clade membership and eBurst analysis highlighted 4 identical genotypes (*MP1b*, *ZWF1b*, *AAT1a*, *AAC1*) for DST 392 (P4) and DST 2130 (P6) in clade 4, and 4 identical genotypes (*ADP1a*, *MP1b*, *SYA1*, *ZWF1b*) for DST 2125 (P2) and DST 2129 (P5) in clade 2 with both pairs of DSTs identical at a single genotype (*MP1b*). Together these genetic divisions and groupings strongly confirm independent origins for these isolates with no shared source of infection for these patients (Table 2, Fig 1 and S3 Fig).

### Susceptibility to antifungal drugs

In general, few changes in drug resistance profiles of isolates were noted over the time course of the study. Most isolates were resistant to both azoles tested from the start of the study period with MIC values above published breakpoints. The MIC testing revealed similar susceptibility profiles within a doubling dilution range across multiple isolates from the same swab site and across multiple swabs at multiple body sites and time points with the exception of two sets of voriconazole values. A fourfold increase was recoded in voriconazole MIC for P1 and a fourfold decrease in voriconazole MIC50 occurred between the penultimate and final swab for P3 (data not shown), the MIC90 values for these isolates remained stable across all swabs for the patients. Overall these susceptibility data show MIC values for strains of a consistent and relatively highly resistant phenotype with no notable acquisition of increased resistance during sustained chronic carriage over the study period (Table 2).

### SNP frequency distribution

To assess the distribution of SNPs across MLST sequences, SNP genotype frequencies were computed for a non-redundant dataset of 2867 isolates taken from the *C*. *albicans* MLST database (Dataset A), and used as a normalising dataset for 6 further datasets with distinct epidemiological characteristics (Datasets B-G), including 6 individual DSTs identified herein (Dataset C).

**Fig 2:**
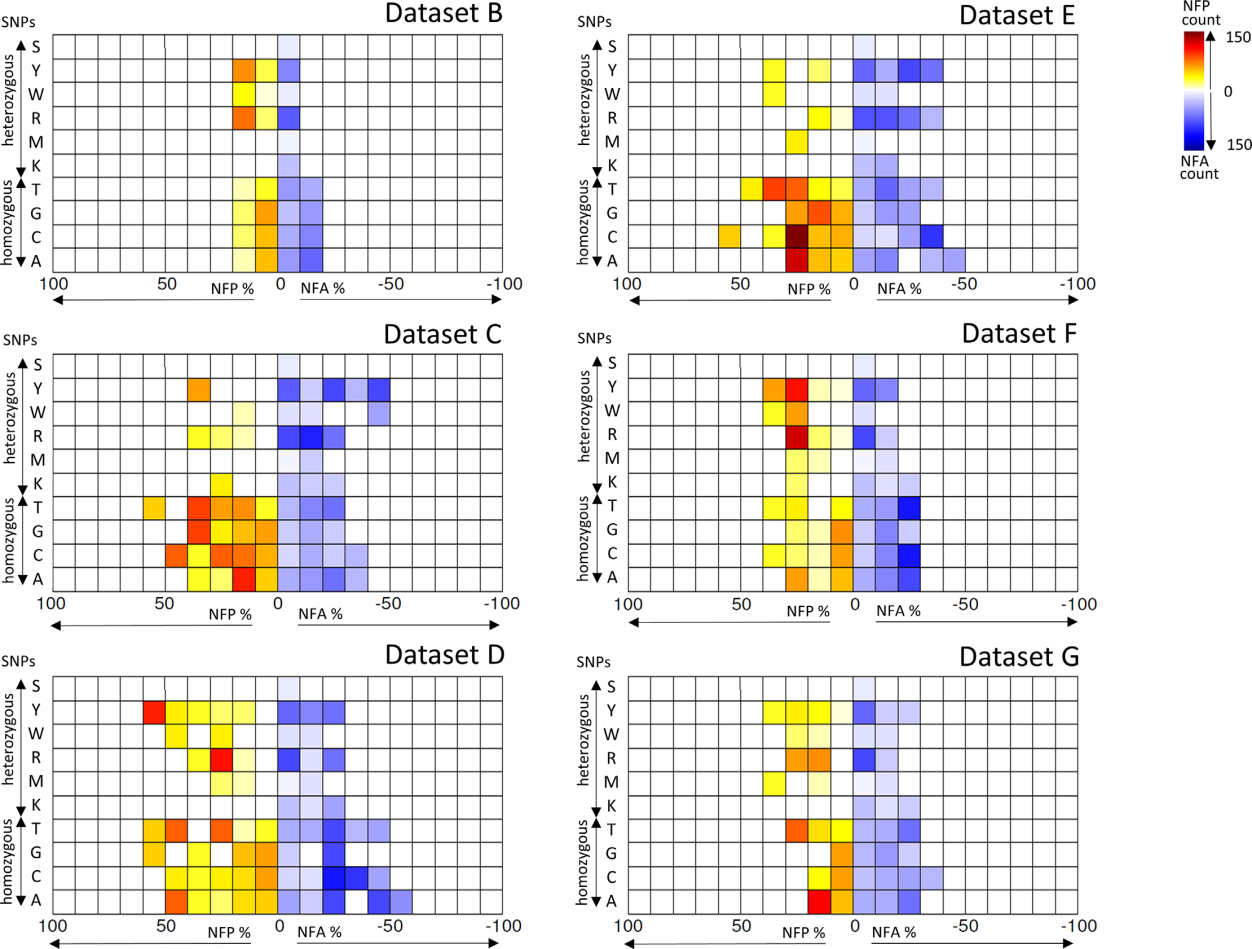
Normalised frequency distribution (NFD) of 6 comparator datasets. NFD heat-map of frequency differences for six datasets (B-G) normalised against a non-redundant dataset of 2867 isolates curated at the central C. albicans database (Dataset A).

Patterns of SNP frequencies for datasets B-G normalised against Dataset A were visualised in NFD plots to identify hotspots of frequency differences (Fig 2 and S5 Fig). Of particular interest were increased heterozygous normalised frequency absence (hetNFA) accompanied by increased homozygous normalised frequency presence (homNFP), as these SNPs are indicative of LOH events. Interestingly, Dataset C and Dataset E, both derived from isolates obtained from patients with autoimmune conditions, displayed the most pronounced elevated hetNFA and homNFP (Fig 2) resulting in the most negative LOHscore of these datasets (S2 Table). Conversely, isolates obtained from patients with oral and vaginal carriage (Dataset D and Dataset G respectively) exhibit levels of greater heterozygosity, while those of blood stream origin and those of a range of clinical origins (Fig 2, S5 and S6 Figs) exhibited a more balanced distribution (Fig 2 and S5 and S6 Figs). We conclude therefore that these isolates were genetically stable and consistent across different body sites, although a number of LOH events had occurred in the past.

## Discussion

The underlying CMC condition promotes susceptibility to *Candida* carriage and infection and we wished to determine whether such patients commonly exchanged *Candida* isolates or were subject to serial colonisation. We therefore sequenced 42 *C*. *albicans* colonies for MLST from 6 unrelated CMC patients treated at the same hospital, and multiple colonies were typed from swabs taken from multiple body sites across multiple time points over a 17 month period (Table 1 and S1 Fig). A single stable DST was identified for all isolates of each individual patient and no patient yielded isolates with the same DST. These data demonstrate clonal and persistent DSTs, stable over time and identical between multiple infection sites with no shared origin or common source of infection for isolates from these patients. Isolates exhibited elevated azole resistance but no acquisition of resistance was notable within serial isolates from individual patients. However, changes in susceptibility for voriconazole observed at MIC50 but not MIC90 among isolates from patients 1 and 3 hint towards the potential presence of differentiated sub-populations genetically indistinguishable through MLST but that may harbour mutations conferring differences in azole resistance, these do not strongly represent an acquired resistance (Table 2). Additionally, we investigated the landscape of MLST genotypes to highlight differential frequency patterning of homozygous and heterozygous SNPs, these data suggest increased levels of past LOH events for persistent and azole resistant colonisers of patients with autoimmune disorders (Fig 2).

The general stability and persistence of *C*. *albicans* populations has been highlighted previously in MLST studies using isolates taken at serial time points in a range of clinical settings [32–34]. The few MLST studies of isolates causative of CMC have taken this approach and have observed over time occasional MLST micro-variations and associated mutations at drug resistant loci conferring acquired azole drug resistant phenotypes [45,46]. Azoles, are the largest family of antifungal drugs and are popular due to their safe oral profiles, yet there are a number of mechanisms underlying antifungal resistance to azoles [52–57]. Our data identifies existing elevated azole resistance within persistent and clonal colonisers of CMC patients and is congruent with previous studies of CMC using MLST.

Genetic diversity among *C*. *albicans* populations can increase through rare recombination events during cryptic mating and as a result of associated concerted chromosome loss following transient aneuploidy [58,59]. However, more frequently genetic variation is driven within populations by LOH [28–31]. MLST consistently identifies LOH rather than strain replacement or larger scale micro-evolutionary changes as driving the generation of new strain types [30–34]. Recent genome wide studies have highlighted the importance of LOH in the genetic plasticity of *C*. *albicans*, using lab strains to dissect the role of abiotic stressors to induced LOH [29]. Indeed LOH plays a key role along with SNP and aneuploidy variations in the evolution of antifungal resistance among clinical isolates [31], and is coupled with mutations at several mediators of azole resistance including *TAC1*, *CDR1/2*, *ERG11* and *MRR1(MDR)* [60,52]. Specifically these molecular mechanisms are the origin of acquired azole resistance among APECED CMC patients in two separate studies that identify MLST LOH mutations along with *ERG11* and *TAC1* derived overexpression of *CDR1/2* [45,46].

It is well recognised that azole resistance develops with long-term use and is often seen in CMC patients who are on anti-fungal treatment for life. Patients with the PID CMC have an underlying genetic immune defect and are very rare, the incidence estimated to be 1:100000. To date, less than 230 patients with GOF-STAT1 mutations have been reported world-wide (Anne Puel, personal communication, manuscript in preparation). As the mutation was identified only recently [3], there is very little longitudinal data, and no data regarding *Candida* spp. strains and sensitivity in these patients. We report no consistent changes in MIC, but observe that MICs for azoles of most isolates were above the reported breakpoints suggesting they were already resistant at the beginning of the period of survey. Some minor changes in voriconazole were noted; in one case a small increase in MIC was seen whilst in another a decrease was observed. We conclude that no marked changes in antifungal drug sensitivity occurred over the 17 months of the survey.

To our knowledge, analysis of SNP frequencies reported here has not been applied previously to *Candida* spp. MLST data in this way. We have developed and implemented a novel and scalable analytical strategy that highlights the potential for MLST data to identify hallmarks of past LOH events from large scale MLST datasets. Interestingly these data suggest indicators of elevated levels of past LOH events are found among *C*. *albicans* isolates from CMC and autoimmune patients in comparison with oral, vaginal and blood stream carriage. Further studies implementing similar analyses with larger datasets may find these signatures of LOH to be synonymous with the increased azole resistance prevalent among autoimmune infection types.

## Conclusions

MLST studies of *Candida* spp. have targeted specific clinical groupings such as nosocomial infections [35,36], periodontitis [40], recurrent bloodstream infection [41,42], cystic fibrosis [39] and genital candidiasis [44]. Although *C*. *albicans* MLST studies have repeatedly reported clade 1 isolates as the most commonly encountered clade and one associated with an increased ability to evolve and to cause infection, repeated attempts to draw further clade associations with clinical classes of infection and with virulence properties have been unsuccessful [43,61]. Similarly, although some geographical enrichment for *C*. *albicans* clades has been observed, MLST is limited in its ability to describe an in-depth evolutionary history of *C*. *albicans* geographic population structure [30,62–64]. This is partly because MLST clades consider only a few hundred mutations in a reductive manner and use phylogenies that ignore the potentially informative properties of mutation frequency dynamics within and between populations. *C*. *albicans* exhibits largely clonal populations yet is capable of large scale genetic plasticity that can rapidly confer selective advantages such as resistance phenotypes. Population distribution analyses for *C*. *albicans* is also made more challenging in an era where individuals commonly travel internationally and may acquire strains that are not normally indigenous.

Our data demonstrates the frequency distribution of SNPs among *C*. *albicans* MLST datasets can provide useful biomarkers such as indicators of lost heterozygosity as a proxy for azole resistance. We detect this signal among isolates colonising CMC patients and suggest this is as a hallmark of selection for resistance in the absence of functional mucosal immunity. Similarly, genetic signatures of conserved heterozygosity may be evinced among commensal isolates of oral, vaginal and GI mucosae where mating and recombination may be more frequent. Using these approaches to molecular epidemiology of *C*. *albicans* isolates may provide improved methods to distinguish between clinical and geographical cohorts beyond using clades alone. Future studies implementing higher throughput genome wide sequencing, together with more sophisticated models for population dynamics of medically relevant yeasts [65–67], will improve our understanding of the population dynamics of fungal infection in diverse epidemiological arrangements and inform our approach to preventative and therapeutic treatments and strategies.

## Supporting Information

S1 Fig Sampling Time Line. A time line of sampling for 42 *C*. *albicans* isolates from 6 CMC patients. Patient number, number of isolates and isolate source are indicated in black, month of sampling appears in blue, red line break indicates turn of the year.
(TIF)

S2 Fig SplitsTree of DST profiles from 6 CMC patients. Distance annotations appear on branches, MLST clades memberships appear at tips for single DSTs, blue and yellow ovals indicate clade 2 and clade 4 isolates respectively.
(TIF)

S3 Fig Alignment and UPGMA phylogeny. Multiple sequence alignment of known MLST SNP base positions from the 6 DSTs from the 6 CMC patients, and UPGMA phylogeny constructed from the same alignment.
(TIF)

S4 Fig Dataset Summary statistics. Clade and genotype membership for Dataset A and summary ratios for datasets B-G (lower panel).
(TIF)

S5 Fig Normalised Frequency Distribution (NFD) Plots. Individual DST NFD plots of isolates identified in the present study (Dataset C).
(TIF)

S6 Fig Principal Component Analysis (PCA). PCA of NFD summary data from Table 2. Autoimmune datasets C and E (green), oral and vaginal carriage datasets D and G (gold), and larger individual isolate datasets B and F (blue) are included (left), and together with individual DSTs from data set C identified in the present study (black and numbered by DST).
(TIF)

S1 Table Primer table. Table of primers used to generate sequence data for *C*. *albicans* MLST typing.
(DOCX)

S2 Table NFD Summary statistics. Table of NFD summary statistics for individual DSTs identified in the present study (dataset C) and for datasets B-G. Loss of heterozygosity score is calculated as (LOHscore) = (hetNFA + homNFP)—(homNFA + homNFP).
(DOCX)
