# Supplementary figures and images for: Clonal Strain Persistence of Candida albicans Isolates from Chronic Mucocutaneous Candidiasis Patients
Source: PLoS One. 2016 Feb 5;11(2):e0145888. doi: 10.1371/journal.pone.0145888 (PMC4743940; doi:10.1371/journal.pone.0145888)

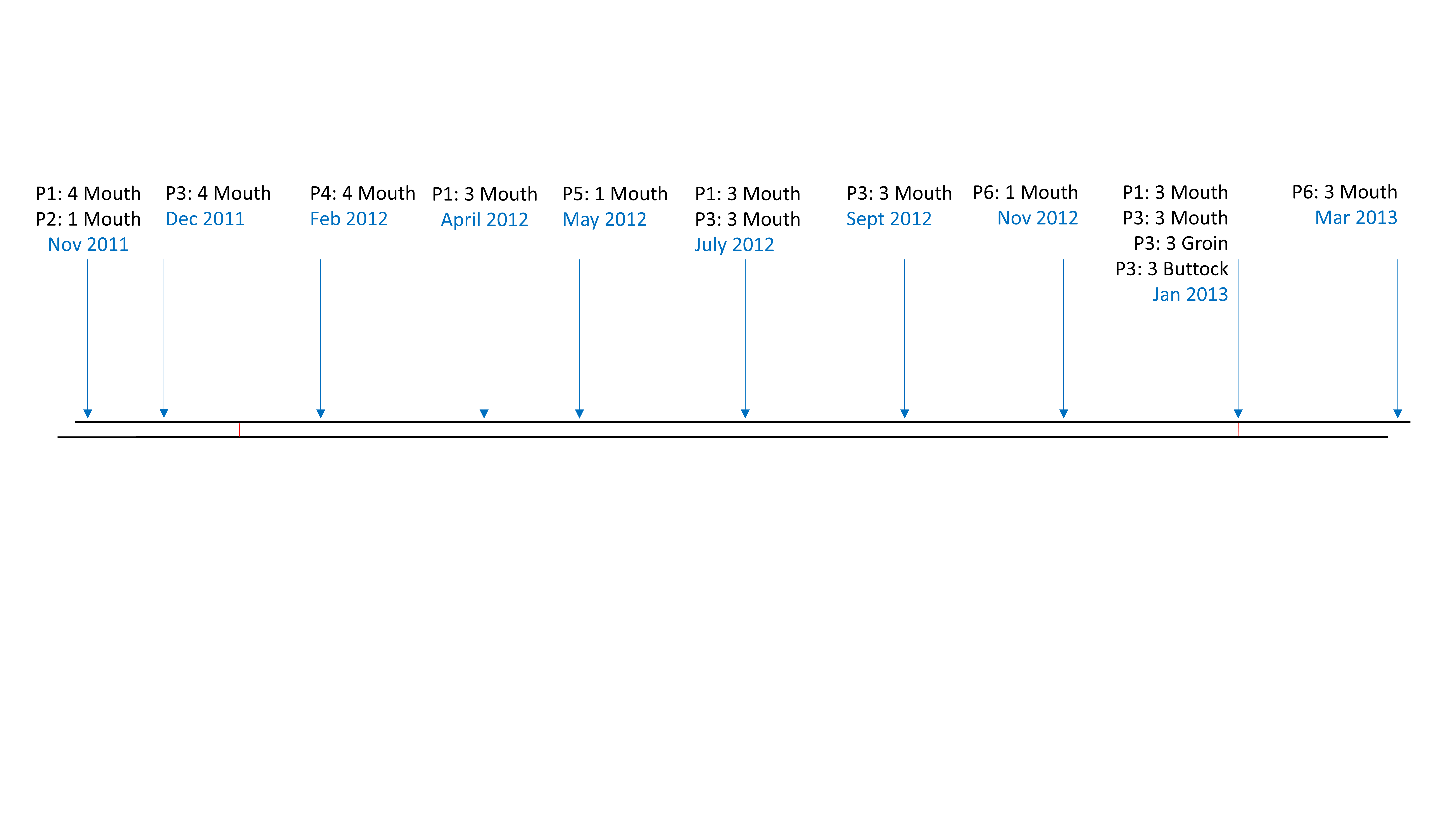

Supplement: S1 Fig — A time line of sampling for 42 C. albicans isolates from 6 CMC patients. Patient number, number of isolates and isolate source are indicated in black, month of sampling appears in blue, red line break indicates turn of the year. (TIF) [file pone.0145888.s001.TIF]

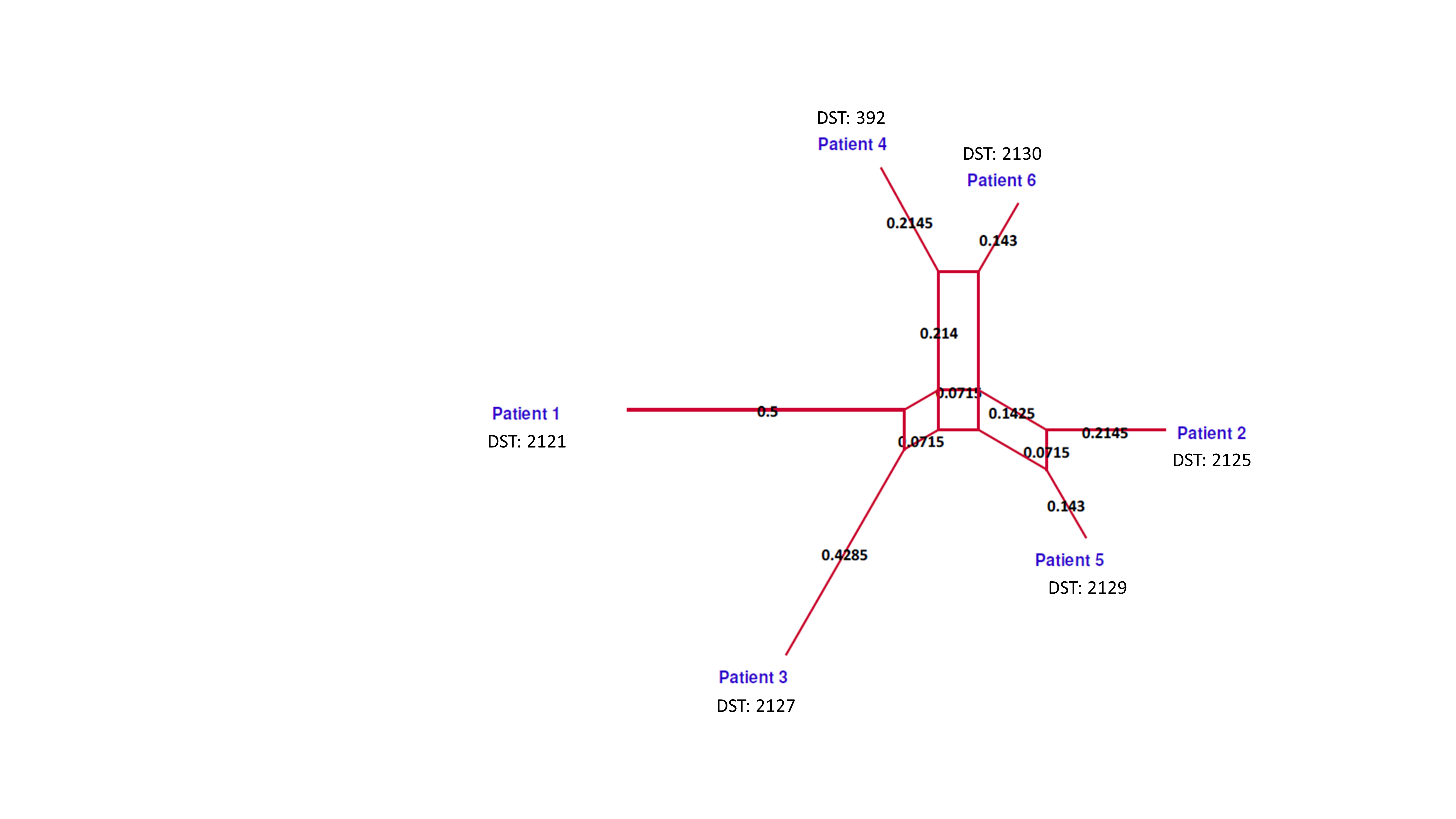

Supplement: S2 Fig — Distance annotations appear on branches, MLST clades memberships appear at tips for single DSTs, blue and yellow ovals indicate clade 2 and clade 4 isolates respectively. (TIF) [file pone.0145888.s002.TIF]

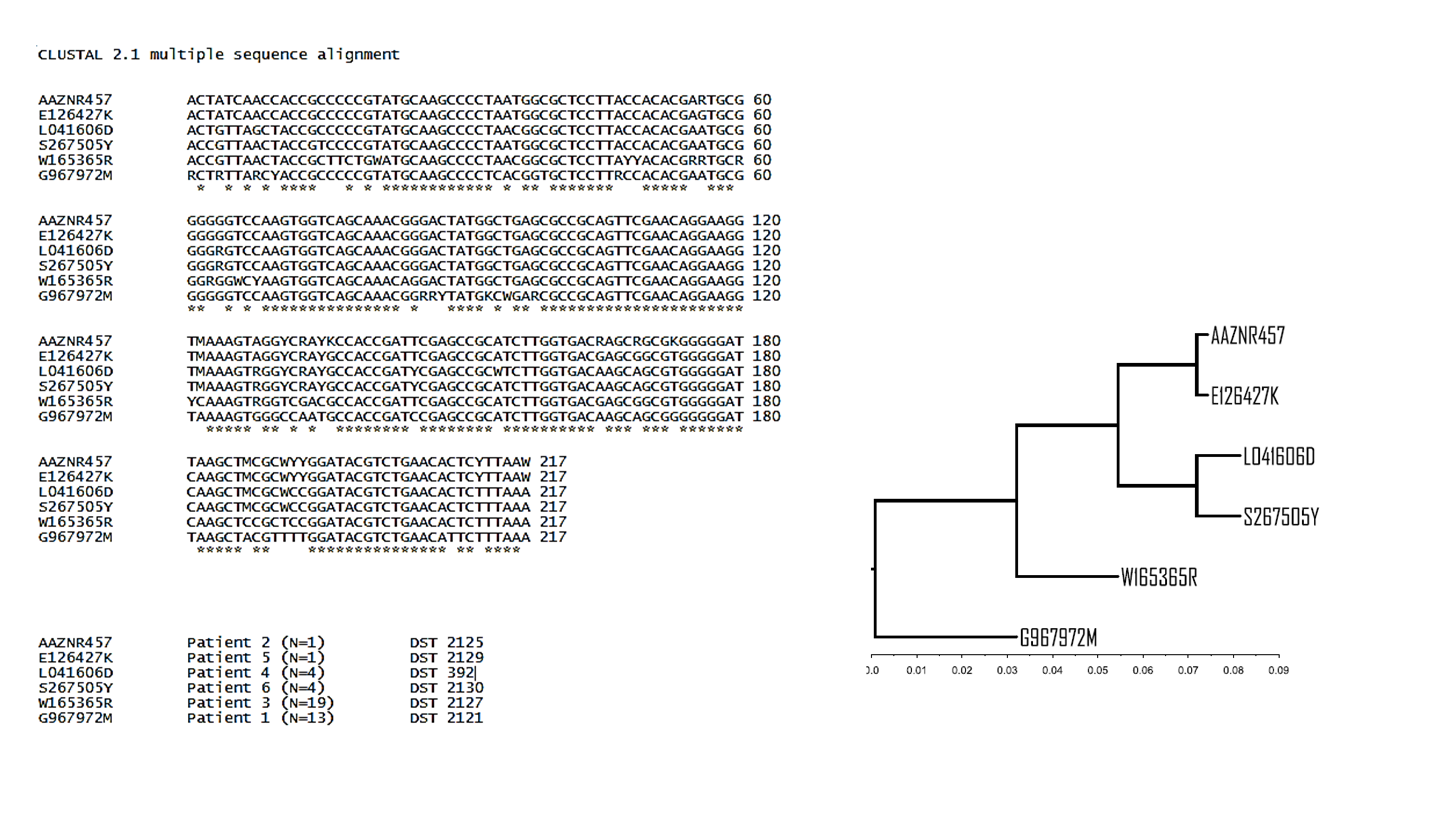

Supplement: S3 Fig — Multiple sequence alignment of known MLST SNP base positions from the 6 DSTs from the 6 CMC patients, and UPGMA phylogeny constructed from the same alignment. (TIF) [file pone.0145888.s003.TIF]

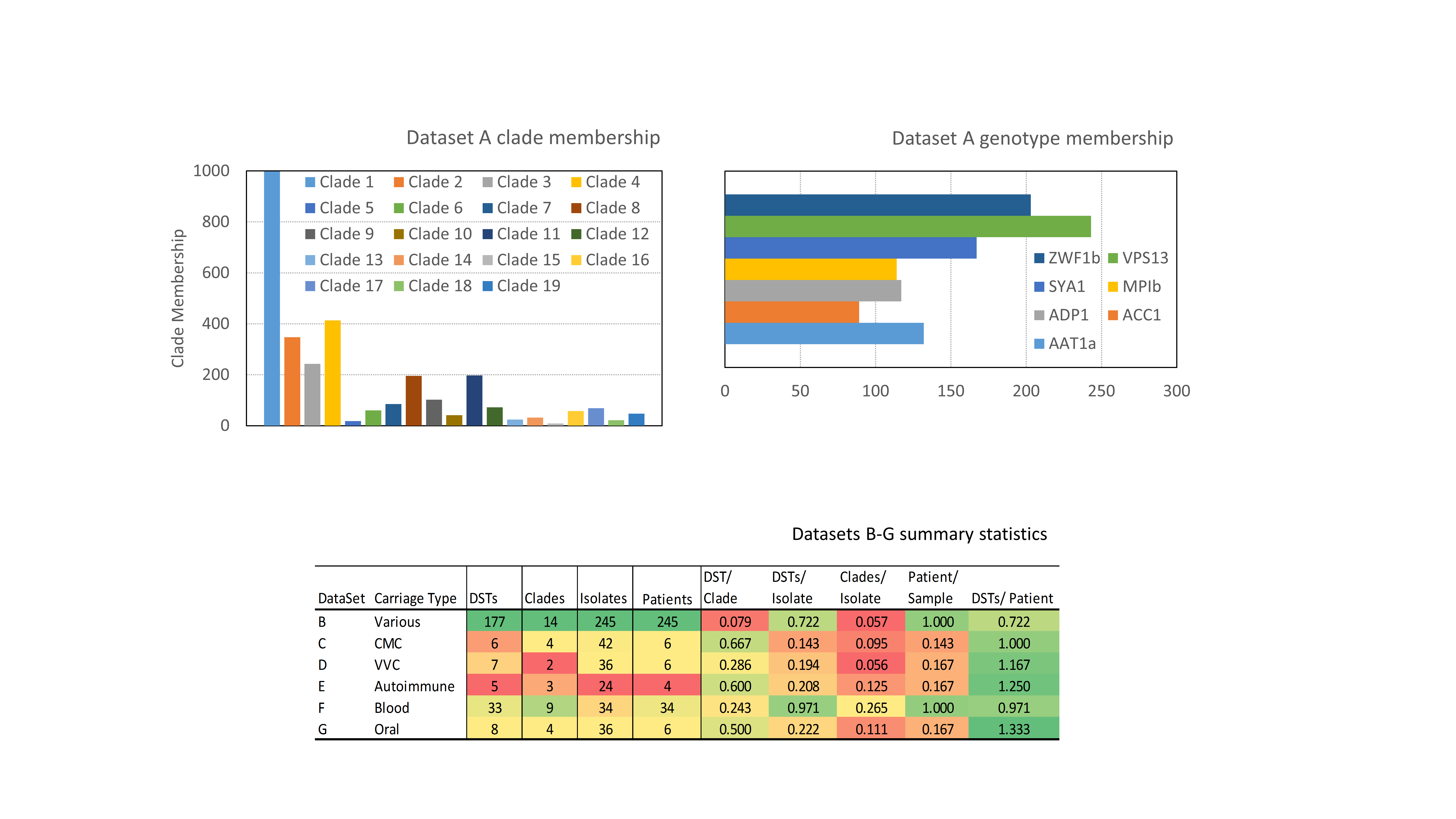

Supplement: S4 Fig — Clade and genotype membership for Dataset A and summary ratios for datasets B-G (lower panel). (TIF) [file pone.0145888.s004.TIF]

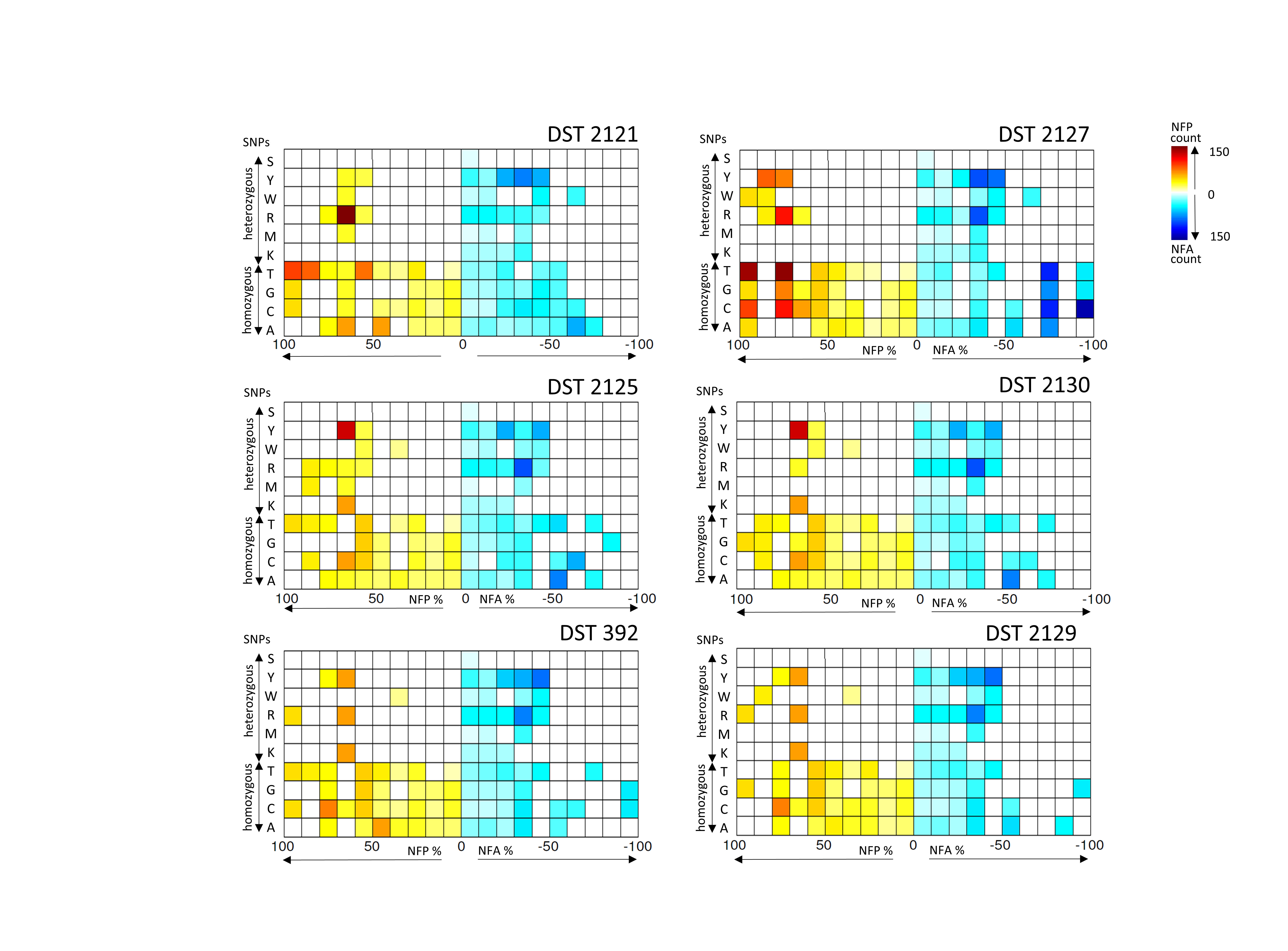

Supplement: S5 Fig — Individual DST NFD plots of isolates identified in the present study (Dataset C). (TIF) [file pone.0145888.s005.TIF]

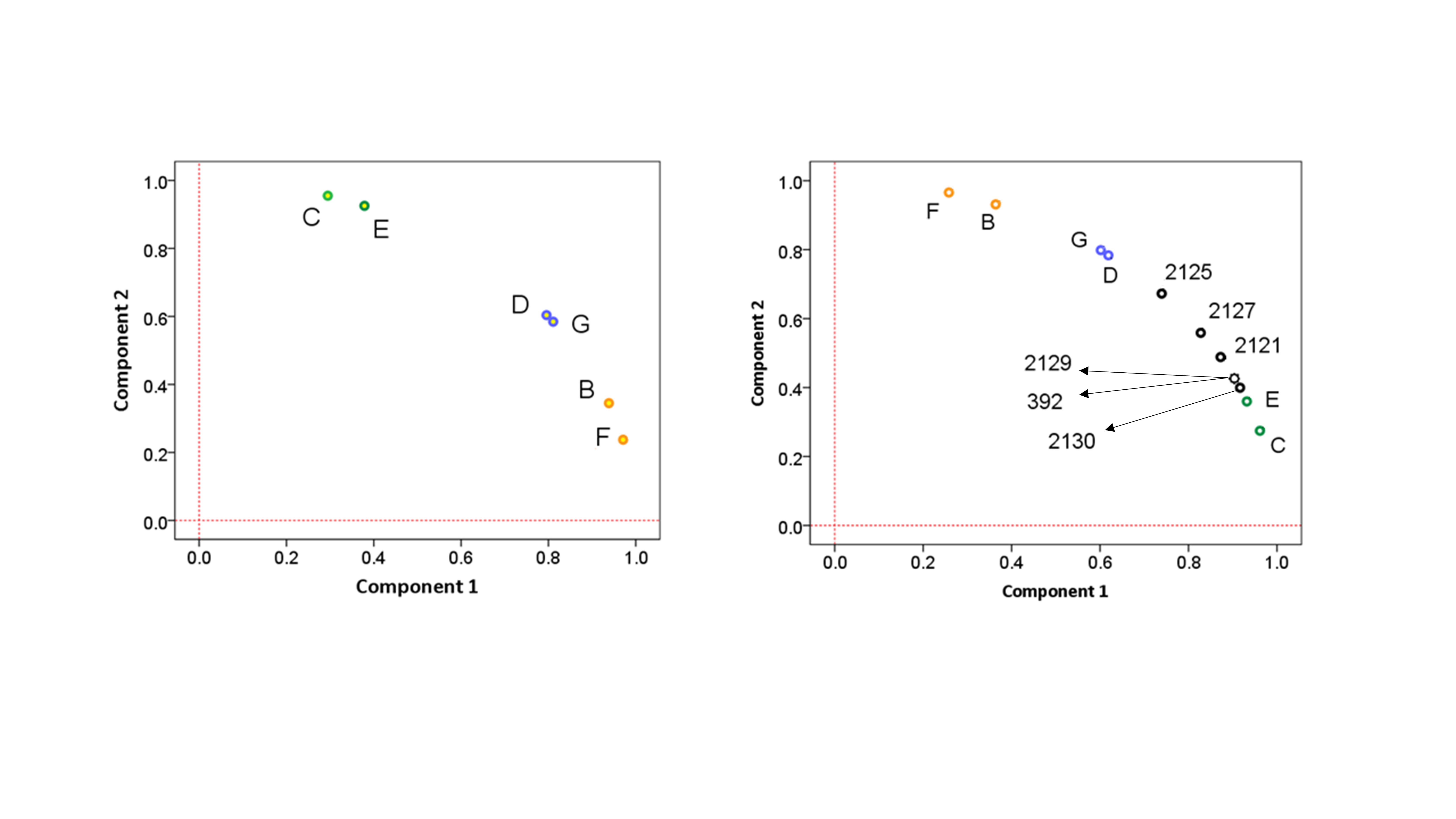

Supplement: S6 Fig — PCA of NFD summary data from Table 2. Autoimmune datasets C and E (green), oral and vaginal carriage datasets D and G (gold), and larger individual isolate datasets B and F (blue) are included (left), and together with individual DSTs from data set C identified in the present study (black and numbered by DST). (TIF) [file pone.0145888.s006.tif]
