# Supplementary material for: Clonal Strain Persistence of Candida albicans Isolates from Chronic Mucocutaneous Candidiasis Patients
Source: PLoS One. 2016 Feb 5;11(2):e0145888. doi: 10.1371/journal.pone.0145888 (PMC4743940; doi:10.1371/journal.pone.0145888)
Supplement: S1 Table — Table of primers used to generate sequence data for C. albicans MLST typing. (DOCX) [file pone.0145888.s007.docx]

| **Locus** | **Amplicon size (bp)** | **Sequenced fragment size (bp)** | **Primer** |  |  |  |
| --- | --- | --- | --- | --- | --- | --- |
| AAT1a | 478 | 349 | Fwd 5’-ACTCAAGCTAGATTTTTGGC- 3’ | | | |
|  |  |  | Rev 5’-CAGCAACATGATTAGCCC-3’ | | | |
| ACC1 | 519 | 407 | Fwd 5’-GCAAGAGAAATTTTAATTCAATG-3’ | | | |
|  |  |  | Rev 5’-TTCATCAACATCATCCAAGTG- 3’ | | | |
| ADP1 | 537 | 443 | Fwd 5’-GAGCCAAGTATGAATGATTTG- 3’ | | | |
|  |  |  | Rev 5’-TTGATCAACAAACCCGATAAT- 3’ | | | |
| MPI b | 486 | 375 | Fwd 5’-ACCAGAAATGGCCATTGC-3’ | | | |
|  |  |  | Rev 5’-GCAGCCATGCATTCAATTAT- 3’ | | | |
| SYA1 | 543 | 391 | Fwd 5’-AGAAGAATTGTTGCTGTTACTG- 3’ | | | |
|  |  |  | Rev 5’-GTTACCTTTACCACCAGCTTT- 3’ | | | |
| VPS13 | 741 | 403 | Fwd 5’-TCGTTGAGAGATATTCGACTT- 3’ | | | |
|  |  |  | Rev 5’-ACGGATGGATCTCCAGTCC- 3’ | | | |
| ZWF1b | 702 | 491 | Fwd 5’-GTTTCATTTGATCCTGAAGC- 3’ | | | |
|  |  |  | Rev 5’-GCCATTGATAAGTACCTGGAT- 3’ | | | |

**Table S1: Primer table.** Table of primers used to generate sequence data for *C. albicans* MLST typing
