# Supplementary material for: Clonal Strain Persistence of Candida albicans Isolates from Chronic Mucocutaneous Candidiasis Patients
Source: PLoS One. 2016 Feb 5;11(2):e0145888. doi: 10.1371/journal.pone.0145888 (PMC4743940; doi:10.1371/journal.pone.0145888)
Supplement: S2 Table — Table of NFD summary statistics for individual DSTs identified in the present study (dataset C) and for datasets B-G. Loss of heterozygosity score is calculated as (LOHscore) = (hetNFA + homNFP)—(homNFA + homNFP). (DOCX) [file pone.0145888.s008.docx]

| **DataSet** | **homNFA** | **homNFP** | **hetNFA** | **hetNFP** | **hetNFA +homNFP** | **homNFA +hetNFP** | **LOHscore** | **homNFA stdv** | **homNFPstdv** | **hetNFA stdv** | **hetNFP stdv** |
| --- | --- | --- | --- | --- | --- | --- | --- | --- | --- | --- | --- |
| 2121 | 1262 | 1937 | 1178 | 661 | 3115 | 1923 | -1192 | 30.97 | 47.85 | 35.62 | 42.26 |
| 2125 | 1356 | 1663 | 1054 | 905 | 2717 | 2261 | -456 | 38.34 | 35.42 | 36.05 | 39.82 |
| 2127 | 1892 | 2567 | 1348 | 831 | 3915 | 2723 | -1192 | 66.51 | 70.51 | 43.91 | 41.91 |
| 392 | 1051 | 1818 | 1249 | 640 | 3067 | 1691 | -1376 | 32.81 | 36.97 | 39.92 | 31.27 |
| 2129 | 1121 | 1888 | 1169 | 560 | 3057 | 1681 | -1376 | 33.39 | 33.16 | 37.95 | 36.30 |
| 2130 | 1130 | 1989 | 1260 | 559 | 3249 | 1689 | -1560 | 31.73 | 37.55 | 39.90 | 29.92 |
| B | 348 | 271 | 168 | 231 | 439 | 579 | 140 | 18.18 | 16.14 | 11.67 | 13.85 |
| C | 500 | 1089 | 695 | 184 | 1784 | 684 | -1100 | 19.11 | 33.88 | 24.68 | 10.57 |
| D | 875 | 904 | 465 | 484 | 1369 | 1359 | -10 | 31.83 | 27.43 | 19.15 | 20.89 |
| E | 609 | 1060 | 586 | 173 | 1646 | 782 | -864 | 22.99 | 38.49 | 22.27 | 8.96 |
| F | 639 | 460 | 311 | 488 | 771 | 1127 | 356 | 29.11 | 20.11 | 15.49 | 23.58 |
| G | 488 | 481 | 317 | 322 | 798 | 810 | 12 | 18.91 | 26.40 | 14.66 | 14.21 |

**Table S2: NFD Summary statistics.** Table of NFD summary statistics for individual DSTs identified in the present study (dataset C) and for datasets B-G. Loss of heterozygosity score (LOHscore) = (hetNFA + homNFP) - (homNFA + homNFP).
